# Implicit Multisensory Associations Influence Voice Recognition

**DOI:** 10.1371/journal.pbio.0040326

**Published:** 2006-09-26

**Authors:** Katharina von Kriegstein, Anne-Lise Giraud

**Affiliations:** 1 Department of Neurology, Johann Wolfgang Goethe University, Frankfurt am Main, Germany; 2 Wellcome Department of Imaging Neuroscience, Functional Imaging Laboratory, London, United Kingdom; 3 Département d'Études Cognitives, Ecole Normale Supérieure, Paris, France; National Institute of Mental Health, United States of America

## Abstract

Natural objects provide partially redundant information to the brain through different sensory modalities. For example, voices and faces both give information about the speech content, age, and gender of a person. Thanks to this redundancy, multimodal recognition is fast, robust, and automatic. In unimodal perception, however, only part of the information about an object is available. Here, we addressed whether, even under conditions of unimodal sensory input, crossmodal neural circuits that have been shaped by previous associative learning become activated and underpin a performance benefit. We measured brain activity with functional magnetic resonance imaging before, while, and after participants learned to associate either sensory redundant stimuli, i.e. voices and faces, or arbitrary multimodal combinations, i.e. voices and written names, ring tones, and cell phones or brand names of these cell phones. After learning, participants were better at recognizing unimodal auditory voices that had been paired with faces than those paired with written names, and association of voices with faces resulted in an increased functional coupling between voice and face areas. No such effects were observed for ring tones that had been paired with cell phones or names. These findings demonstrate that brief exposure to ecologically valid and sensory redundant stimulus pairs, such as voices and faces, induces specific multisensory associations. Consistent with predictive coding theories, associative representations become thereafter available for unimodal perception and facilitate object recognition. These data suggest that for natural objects effective predictive signals can be generated across sensory systems and proceed by optimization of functional connectivity between specialized cortical sensory modules.

## Abstract 2

This study illustrates that recognition of natural objects under conditions where only one sensory modality is available can rely on implicit access to multisensory representations of the stimulus.

## Introduction

Our senses sample different types of physical information from each object we encounter in the natural world. Part of this multisensory information is redundant because some object properties are conveyed through several of our sensory modalities: Judging gender or age of a person for instance is almost as easily derived from listening to the voice as from looking at the face of a person, because vocal sounds are in part determined by face and neck spatial configuration [1–4]. In unimodal presentation of natural objects, missing information that normally comes from the other senses might automatically be reconstructed. Hearing a voice on the telephone is a typical situation where evoking the corresponding face may help to identify the speaker. It is unknown how these multimodal features, e.g. voices and faces, are combined by the brain and whether implicit access to multisensory representations entails a behavioural benefit in unimodal recognition. If this was the case, this would speak to predictive coding and other forward models of brain functioning, which assume that higher-order neuronal levels influence lower processing stages through feedback loops [5–10]. This study hence tackles the neural expression of predictive coding across sensory modalities and examines its relevance for unimodal processing.

Multimodal association is classically viewed as a hierarchical mechanism by which visual and auditory information converge onto supra-modal representations [11]. Accordingly, psychological models of person recognition postulate that voice, face and other person related information, e.g. name, merge in higher level supramodal person representations [12–16], classically referred to as Person Identity Nodes (PINs [16]). This convergence of information might take place in the anterior temporal lobe [17,18], as lesions to this region abolish the ability to recognize persons irrespective of the input modality. Yet, physiological studies reveal crossmodal effects at very early processing stages [19–22]. Responses to vocalizing faces have been observed in monkey [22] and human [20,21] auditory cortex, indicating that already sensory cortices are responsive to crossmodal voice and face information. The level at which multimodal information is first combined is an important issue as only *early* multisensory associations may significantly and reliably influence unimodal person recognition. Functional neuroimaging studies in humans show that voices of familiar speakers activate the fusiform face area (FFA, [23]) via the temporal voice areas (TVA, [24]), which supports early interactions between cortical sensory modules [25]. It remains unclear, however, whether this neural effect entails a behavioural benefit for voice recognition by granting access to early distributed multisensory representations, e.g. voice-face sensory templates devoid of contextual person-related information, and how these representations neurally interact with supramodal person recognition levels, e.g. PIN.

To address these questions we investigated neural and behavioural changes induced by new associations with voices in the absence of prior knowledge about the speaker. We further explored whether the induced changes in behaviour and neural activity measured with functional magnetic resonance imaging (fMRI) require sensory redundancy across modalities, i.e. that both modalities convey the same object features, or whether these changes also occur after association of arbitrary multimodal stimulus combinations, i.e. when each modality convey independent object features. Our experimental paradigm involved two groups of participants. One group learned to associate voices with faces. Voices and faces provide congruent and sensory redundant information about gender, ethnic group, age, size, and identity [1–4]. Voice-face learning therefore corresponds to a multisensory association, in which the associated stimulus characterizes the person as a physical object and provides redundant sensory information about the person. The second group learned to associate voices with names. In contrast to faces, names are arbitrarily related to voices. Given the same gender and ethnic background, almost any name can be associated with any voice. This voice-name learning, therefore, corresponds to a congruent but arbitrary multimodal association. Voice-name association, however, balances voice-face learning, because matching names with voices is about as frequent in everyday life as matching faces with voices, and it additionally controls for familiarization with voices throughout the experiment and potential expertise effects [26,27].

Associative learning of voice-face and voice-name does not only differ in that the latter are arbitrarily related, but also in that voices and names do not originate from the same source. Although both associations establish strictly equivalent statistical connections between the two stimuli, voices and faces are by nature not dissociable, in the sense that (ventriloquism aside) natural voices are always produced by faces and, more importantly perhaps, co-modulated over time. We used ring tones and cell phones as additional control stimuli to address the importance of a common physical source for sounds and visual stimuli in the shaping of early multisensory associations and subsequent unimodal sound recognition. Although ring tones and cell phones relate to a unique ecologically valid multimodal source, their association is arbitrary. Any ring tone can be wired to any cell phone and therefore cannot predict the model or brand of a phone.

**Figure 1:**
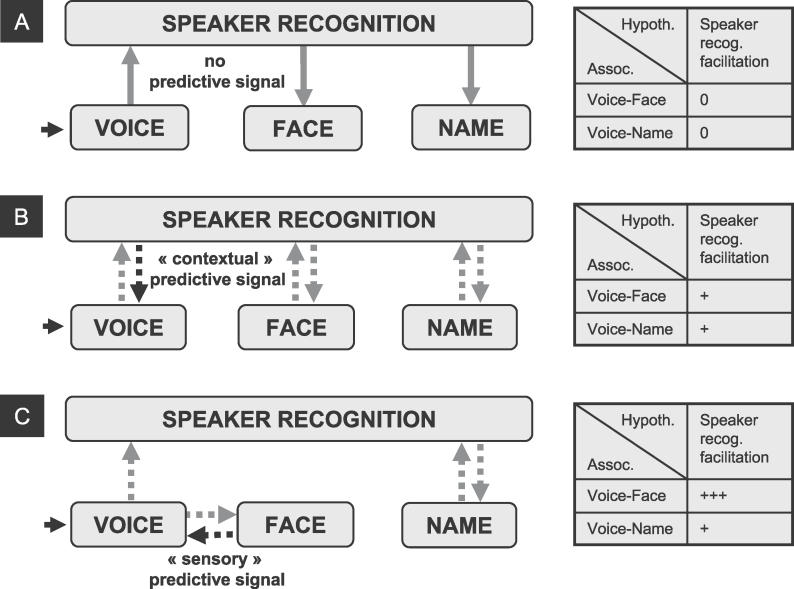
Models of Functional Coupling between Voice and Face Modules Operating during Unimodal Speaker Recognition (after Associative Learning) (A) Person identity recognition models [12,16]. (B) Adaptation of (A) with reciprocal interactions between modules. (C) Adaptation of (B) with direct reciprocal interactions between sensory modules. The plain arrow indicates a bottom-up signal that affords speaker recognition. Dotted arrows indicate predictive signals (black, informing voice module). Hypotheses related to each model are indicated in tables. Hypoth: hypothesis, Assoc: associative learning, Recog: recognition.

This experimental setting allows us to distinguish between three competing models of voice-face interactions during unimodal voice recognition (Figure 1). In the first model, speaker identity is directly retrieved and neural interactions between TVA and FFA [25] denote visual associations as a by-product of person recognition (Figure 1A). In this case, specific voice-face associative learning should have a small impact on the neural TVA-FFA coupling and should not facilitate subsequent speaker recognition. In the second model, speaker recognition is facilitated by implicit access to any contextual information about the speaker, faces or names, depending on the group tested (Figure 1B). In this case, voice-face and voice-name learning should strengthen connectivity between TVA and FFA, and TVA and regions responding to written names, respectively. This strengthening of functional connectivity would involve a supramodal relay stage. Importantly, both types of learning should equally facilitate subsequent unimodal speaker recognition. In the third model (Figure 1C) speaker recognition is strongly facilitated thanks to direct access to an internal multisensory template implemented in a voice-face feedback loop. If the latter model is valid we should observe a strong TVA-FFA neural coupling and speaker recognition should be facilitated only by previous voice-face association, but not by previous voice-name association (see Figure 1 for behavioural hypotheses).

## Results

**Figure 2:**
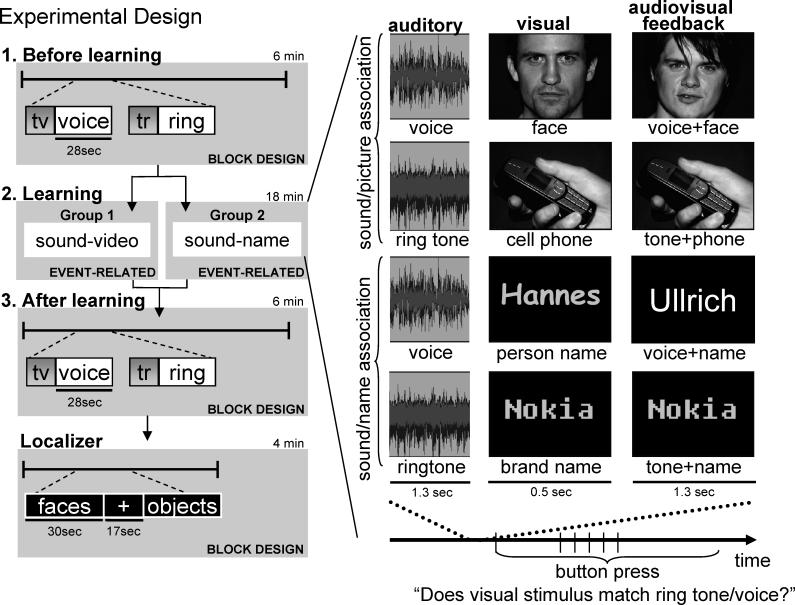
Experimental Design (Left) Two unimodal auditory sessions (Part 1 and Part 3), during which participants recognized either a target voice (tv) or target ring (tr) tone among different voices or ring tones, were carried out before and after learning to associate auditory stimuli with corresponding videos or written names. The two types of learning (Part 2) were performed by separate groups of participants. One group (*n* = 14) learned voice-face and ring tone-cell phone associations (sound-video group), while the other group (*n* = 15) learned voice-name and ring tone-brand name associations (sound-name group). All participants participated in a face area localizer experiment at the end of the protocol, in which they passively viewed pictures of different faces and objects presented in blocks. (Right) Detail of the learning protocol. Participants were requested to match a voice or a ring tone with a picture or a name and gave their response by key-press. Feedback of the correct association (videos in the sound-video group and sound together with the name in the sound-name group) was immediately given after each trial so that the participants progressively learned the correct association.

The experiment had three parts (Figure 2). In Part 1, all 29 participants listened to and identified auditory signals—voices and ring tones—while neuronal activity was assessed using block-design fMRI. In Part 2, participants learned to associate these voices and ring tones with visual stimuli. One group of 14 participants learned voice-face and ring tone-phone associations (see Videos S1 and S2 for examples of the videos used for learning) whereas another group (15 participants) learned voice-person name and ring tone-brand name association while neuronal activity was assessed using an event-related fMRI; in Part 3, the auditory-alone experiment was repeated. The unimodal auditory parts (Part 1 and 3) were analyzed independently from the multisensory learning part (Part 2). Functional neuroimaging and behavioural results corresponding to auditory recognition sessions before and after learning, and to the multimodal learning part are described separately in the following.

### Unimodal Voice Recognition

#### Effect of multisensory experience on brain activity during voice recognition.

**Figure 3:**
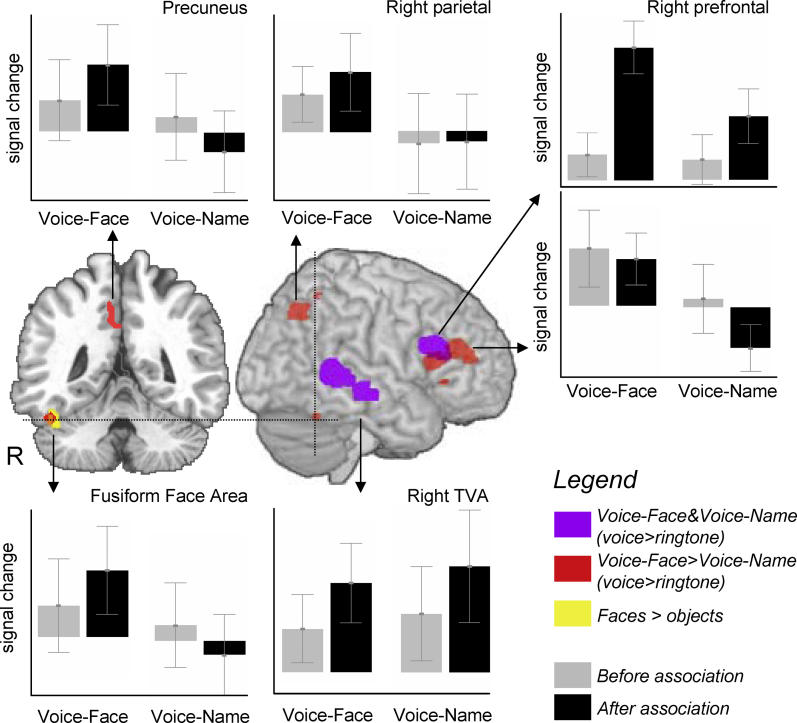
fMRI Activity in Response to Voice Recognition The surface rendering shows responses during voice recognition compared with ring tone recognition (*n* = 29). In purple: after both voice-face and voice-name association learning (*p* < 0.001 uncorrected); In red: after voice-face more than after voice-name learning (*p* < 0.001 uncorrected). A coronal section through the FFA shows the overlap of the crossmodal activation by voices (in red) with the responses to faces presented visually during the face localizer experiment (yellow). Plots of signal change in response to voice recognition contrasted with ring tone recognition are displayed for each responsive brain region. Error bars represent 95% confidence interval of the mean.

A whole brain analysis was carried out to identify brain regions where responses to voices, relative to ring tones, were enhanced after multimodal experience. Contrasting activity for voice recognition (irrespective of whether voices were previously associated with faces or names) with that for ring tone recognition (irrespective of whether ring tones were previously paired with cell phones or brand names of cell phones) resulted in activations in the right prefrontal cortex (BA44/45) and the right posterior superior temporal sulcus (TVA) (Figure 3, purple blobs). These findings concur with data showing that multisensory learning generally enhances activity in areas that are involved in unimodal sensory processing [28].

#### Effect of voice-face association on brain activity during voice recognition.

Regions where voice-face association influenced subsequent voice recognition were probed by an interaction between stimulus type (voice, ring tone) and learning group (sound-video, sound-name group). In other words, responses during voice recognition after voice-face association (relative to ring tone recognition after cell phone association) were contrasted with those obtained after voice-name association (relative to ring tone recognition after ring tone-name association). Previous voice-face association enhanced responses to voices in the fusiform cortex, the precuneus, bilateral parietal cortices, and the right prefrontal cortex. These regions have all been previously implicated in the processing of familiar persons, independent of input modality [25,29–31], and by face imagery [32,33].

The coronal section in Figure 3 shows that the response to voices in the fusiform cortex overlaps the FFA, as functionally defined by a separate face area localizer (contrasting activity for pictures of faces against activity for pictures of objects, see Figure 2 and Materials and Methods).

Crossmodal effects in the FFA were further investigated using a region of interest (ROI) approach. The FFA was functionally mapped in each participant using the contrast between visually presented faces and objects. Within this ROI, we confirmed that FFA activation during voice recognition (relative to ring tone recognition) increased only in the group that learned voice-face associations (group contrast, *p* < 0.04, corrected, see plot for FFA in Figure 3). This specificity was obvious at the individual level, as 11 of the 14 participants who learned voice-face associations exhibited a significant effect in response to voices after voice-face learning in the FFA, which was not detected after voice-name learning.

Crossmodal responses to ring tones were also investigated by the interaction between stimulus (voice, ring tone) and learning group (sound-video, sound-name group), i.e. by contrasting activity in response to ring tone recognition after cell phone association (relative to voice recognition after face association) with activity in response to ring tone recognition after ring tone-name association (relative to voice recognition after voice-name association). No response (even at low *p* < 0.01 uncorrected threshold) to ring tones was found in cortical regions responding to visual objects (as defined by the functional localizer).

Crossmodal responses in visual areas responding to written names were investigated for voice and ring tone recognition separately. Contrasting activity in response to voice recognition, after learning names with activity, after learning faces, did not reveal activity (*p* < 0.01, uncorrected) in areas usually responsive to visual word forms [34]. There was also no differential response (*p* < 0.01, uncorrected) in the activity in the visual word forms in response to ring tone recognition after learning brand names in comparison to activity after learning the corresponding cell phones.

In summary, activation of specialized visual cortex in response to auditory input, was only observed after experience of redundant multisensory stimuli (voice-face), but not after experience of arbitrarily related multimodal information (voice-name, ring tone-name), even when the stimulus pair was congruent at the sensory level (ring tone-cell phone).

#### Changes in functional connectivity associated with voice-face learning.

**Figure 4:**
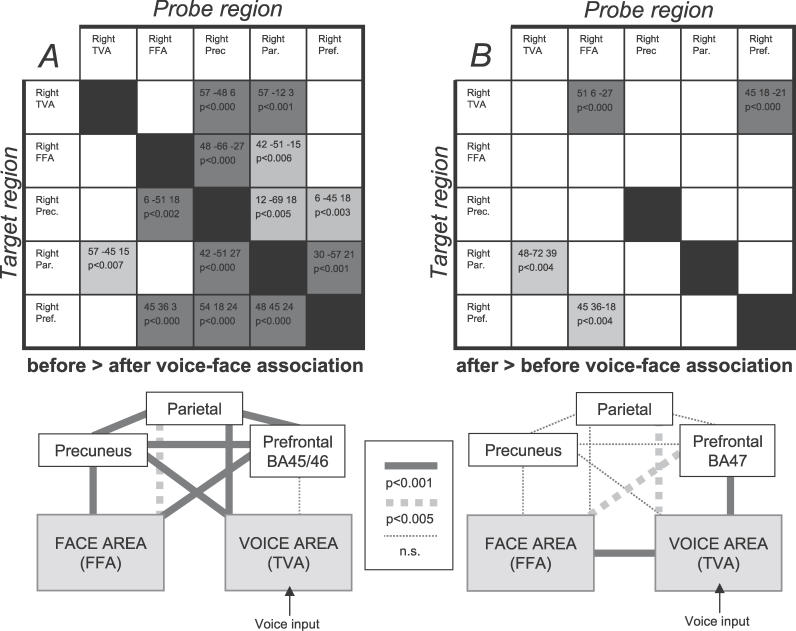
The Impact of Voice-Face Associative Learning on Functional Connectivity All areas shown in red in Figure 3 were included in functional connectivity analyses assessed by means of PPI. In addition, the TVA was included as entry point in the voice recognition network. PPIs probed changes in functional connectivity across regions during voice recognition resulting from learning of voice-face associations. All five regions served both as probes and targets and the results are presented in double entry tables. The colours in boxes indicate the level of statistical significance associated with the hypothesis of enhanced connectivity: dark grey for *p* < 0.001, (uncorrected), light grey for *p* < 0.01, white for non significant, and dark for autocorrelation. Numbers indicate the coordinates (x, y, z in MNI template) of the voxel where maximal correlation was found. All 14 participants who had learned faces in response to voices were included in the analysis. (A) Before voice-face association > after. (B) After voice-face association > before. Figures below tables illustrate the impact of learning on functional connectivity. Enhanced connectivity is represented as dark grey links.

Enhanced functional connectivity between TVA and FFA has been shown during recognition of familiar speaker's voices [25], indicating a direct interaction. Here we investigate whether a brief association between voices and faces of unknown speakers could induce this direct functional connectivity between TVA and FFA. Functional connectivity was assessed using psychophysiological interactions (PPI [35], see Materials and Methods) in a condition-specific manner (voice recognition after voice-face learning contrasted against voice recognition before voice-face learning, and vice versa). Corresponding correlations were computed across the four regions that selectively enhanced their activity in response to voices after voice-face learning (right FFA, right parietal, right precuneus, right prefrontal). TVA was also included in the correlation matrix as it is the major entry point of the functional network recruited during voice recognition. Results are summarized in Figure 4, which depicts functional connections during voice recognition that are significantly stronger before than after learning (Figure 4A) and reciprocally, those that are stronger after learning than before (Figure 4B).

In Part 1 of the experiment when participants recognized a target voice before seeing the corresponding face, functional interactions were significantly stronger than after voice-face learning between the right TVA and the precuneus, right TVA and right parietal cortex, the precuneus and the right prefrontal cortex (BA44/45), the precuneus and the right parietal cortex, the right parietal and the right prefrontal (BA46), and the right prefrontal (BA46) and the FFA. Functional interaction between the FFA and the precuneus was also stronger, at a slightly lower confidence level (*p* < 0.002, uncorrected). The lower panels in Figure 4 schematically indicate how connections were affected by learning. Because participants did not know the voices before the experiment and had no semantic information about the speakers, associative mental activity during voice recognition was limited to forming mental predictions about speakers, e.g. their physical appearance or gender. That such automatic imagery occurred was confirmed by responses to a questionnaire indicating explicit person imagery in eight out of 14 participants during the initial voice recognition experiment (Protocol S1). PPIs do not reveal directional effects, but given a vocal input, mental images of persons could result from neural information circulating among these regions, as previously observed during explicit mental evocation of faces [33].

Functional interactions within this network changed dramatically after voice-face learning. The pattern of connectivity shifted towards stronger coupling between TVA and FFA, and between TVA and the ventral prefrontal cortex (BA47) exclusively. The increase in TVA-FFA coupling after voice-face learning was observed consistently across individuals. Ten of the 11 participants showing significant crossmodal responses in FFA during voice recognition also showed enhanced functional connectivity between the FFA and individually located voice regions (four at *p* < 0.001, five at *p* < 0.01, one at *p* < 0.05, uncorrected).

#### Behavioural impact of voice-face association on voice recognition.

Voice and ring tone recognition performance was assessed during fMRI scanning before and after these auditory stimuli were associated with the videos (face/cell phone) or names (see Figure 2).

**Figure 5:**
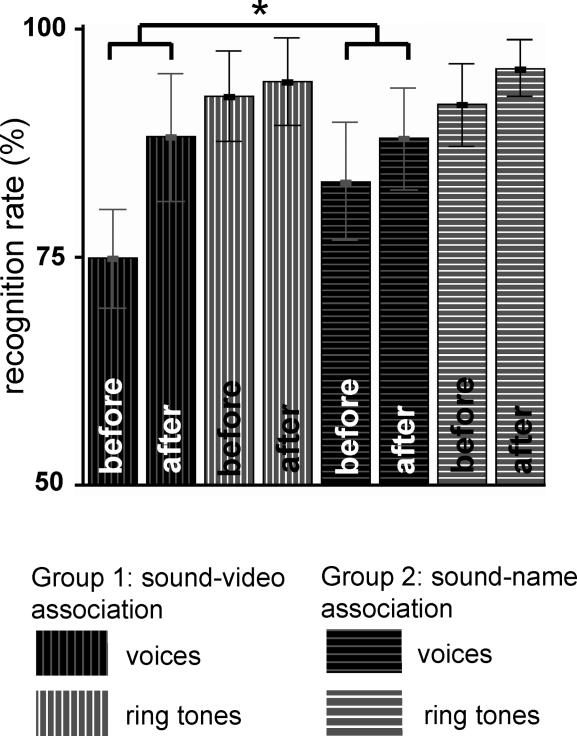
Recognition Scores for both Groups for Voice and Ring Tone Recognition before and after Learning ANOVA on repeated measures revealed a significant crossmodal learning effect in both groups for voice recognition (F[1,27] = 28, *p* <0.0001) and a condition (voice recognition before, voice recognition after learning) by group (sound-video, sound-name group) interaction (F[1,27] = 6, *p* <0.018) reflecting a larger learning effect for voices in the face group than in the name group. For ring tone recognition there was no corresponding condition (ring tone recognition before, ring tone recognition after learning) by group (sound-video, sound-name group) interaction (F[1,27] = 0.4, *p* < 0.6). There was a significant effect of stimulus type before (F[1,27] = 16, *p* < 0.0001) and after learning (F[1,27] = 33, *p* < 0.0001) indicating that voice recognition was overall more difficult than ring tone recognition (post-hoc paired t-tests: before learning t = 5.8, *p* < 0.0001, after learning t = 3.3, *p* < 0.003). All *p*-values are two-tailed. Error bars represent 95% confidence interval of the mean.

Previous studies have shown that associating a face together with a name and a voice improves subsequent naming of the voice presented alone [36]. Here, associating faces with voices improved subsequent recognition of voices by about 14%, whereas associating names with voices only enhanced speaker recognition by 5% (Figure 5). Ring tone recognition also improved by about 5 % after both types of audio-visual learning (cell phone and brand names of cell phones). Selective improvement of speaker recognition after voice-face learning was confirmed by a condition-by-group interaction (Figure 5, see details about the statistical tests used and significance levels in legend).

### Multimodal Learning

#### Brain activations and behavioural scores.

**Figure 6:**
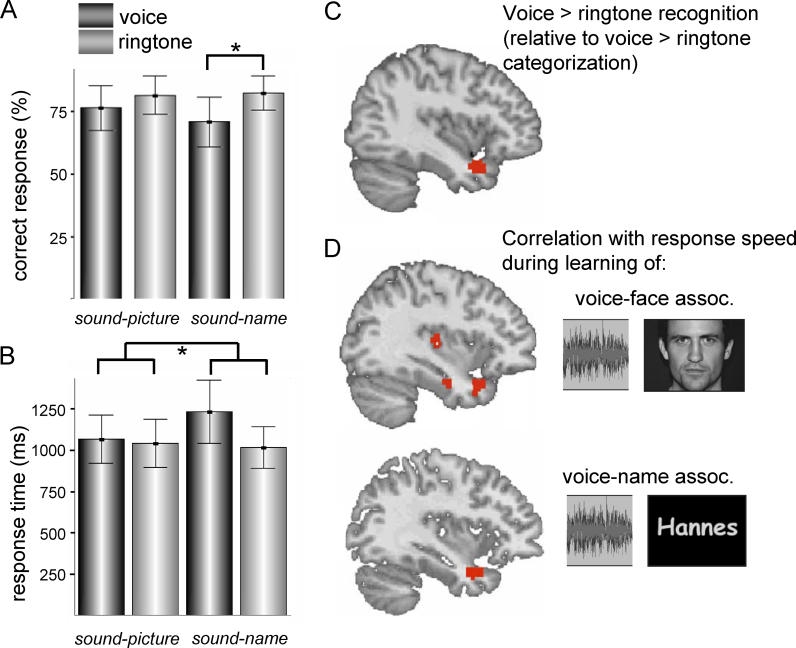
Behavioural Results and fMRI Activity for the Learning Part of the Experiment (A, B) Behavioural measures corresponding to learning are displayed in plots for response correctness (A), and for response time (B). There was no difference between groups regarding correctness. Differences within groups between matching voice and ring tone associations, was significant in the voice-name group only (paired t-test, *p* < 0.03). Voice-name matching yielded longer response times than voice-face matching as revealed by a condition (voice, ring tone) by group (sound-video, sound-name) interaction, ANOVA, F(1,27) = 6, *p* < 0.01). The difference in response time to voices and ring tones was significant in the voice-name group only (paired t-test t = 3.3, *p* < 0.009). All *p*-values are two-tailed. Error bars represent 95% confidence interval of the mean. (C, D) Brain regions involved during voice-face and voice-name learning were analyzed separately (event-related) from the sessions involving auditory recognition. Activity in the anterior temporal cortex which is classically involved in multimodal person recognition was observed during both voice-face and voice-name matching (relative to ring tone-cell phone and ring tone-name matching) when compared with their respective control tasks where the associated stimuli (faces and names, cell phones and brand names) were simply categorized instead of matched with the preceding sounds (C). The same region of the anterior temporal cortex parametrically correlated with the speed of the response in the group performing a voice-face association (*n* = 14; *p* < 0.001, uncorrected) (D) and in the group performing a voice-name association (*n* = 14; *p* < 0.01, uncorrected). When both groups were analyzed together, parametric modulation with response speed was significant (*n* = 28; *p* < 0.001, uncorrected).

The learning part of the experiment was designed as an event-related session and analyzed separately (Figure 2). Behavioural measures obtained while participants were associating voices with visual stimuli support the hypothesis that neural circuits underlying voice-face learning are different from learning compatible but arbitrarily paired stimuli (Figure 6). Although all conditions yielded more than 70% correct responses (Figure 6A), longer response times (Figure 6B) and increased error rates were observed during voice-name associations than voice-face associations. The response was delayed by about 200 ms when participants had to determine whether a name matched a voice, compared to when they had to decide whether a face matched a voice. A significant condition-by-group interaction confirmed that this difference was restricted to voice-face versus voice-name learning, while matching a ring tone with a name was as fast as matching it with a picture of a cell phone (Figure 6A and B; see details about the statistical tests used and significance levels in legend).

Functional MRI data were analyzed to delineate regions where multimodal person-related information converges during person recognition. The anterior temporal cortex is generally involved in multimodal representations of individuals [17,18,29,37]. It did not activate in the contrasts probing joint effects of voice-face and voice-name learning on subsequent voice recognition described in the above sections. It however responded while the participants learned voice-face and voice-name combinations. In particular, activation was higher when participants had to decide whether a face or a name matched a voice (relative to the respective conditions for ring tones), than when they simply categorized visual stimuli (relative to the respective conditions for ring tones) (see Materials and Methods and Figure 6C). Furthermore the activity in the anterior temporal cortex increased with the speed of response to voices during learning (Figure 6D). The more rapidly participants responded whether a face or a name corresponds to a voice, the higher the activity in the anterior temporal cortex.

## Discussion

Our results show that brief exposure to voice-face pairs shape a distributed multimodal sensory representation, which manifests after learning (1) at the neural level as a marked increase in functional connectivity between cortical modules specialized for the processing of each stimulus category (FFA and TVA), and (2) at the behavioural level as enhanced unimodal speaker recognition. This double effect required pairs of stimuli to be sensory redundant, i.e. convey the same object features.

### Changes in Neural Connectivity after Learning

Before voice-face association, the task tapped into a large network comprising the prefrontal and parietal cortices, the precuneus, TVA, and FFA. Similar functional interactions between FFA, precuneus, and parietal and prefrontal regions have previously been seen during explicit mental imagery of faces [33]. It is conceivable that this functional network permits information to circulate between FFA and TVA to sustain visual imagery about a person who is speaking before a one-to-one voice-face association is made. After voice-face learning, all these functional connections weakened and a direct connection appeared between TVA and FFA and between TVA and ventral prefrontal cortex (BA47). In a previous study, FFA activation by voices and functional coupling between TVA and FFA were observed in a developmental prosopagnosic participant, although she was unable to recognize her friends by their faces [38]. It showed that TVA-FFA coupling is dissociated from the ability to mentally evoke a face, but depends on sensory exposure to concurrent voices and faces. Although it is not tenable to generalize from a single case with a developmental disorder, this observation indicates that responses to voices in the FFA result from a “sensory” rather than “contextual” mechanism (Figure 1). These findings have a good physiological plausibility because audio-visual sensory binding has been observed in animals and humans, at even earlier sensory processing stages than voice and face specific processing (for review see [39]) and with latencies that are compatible with a direct influence of inputs in one modality on early responses to stimuli presented in the other [40]. Early crossmodal effects are also backed-up by anatomical studies that used retrograde labelling of monkey visual cortex to show the presence of projections arising from auditory cortex in several cortical layers [41–44].

### Implications of TVA-FFA Coupling for the Functional Role of the FFA

Crossmodal neural activity of the FFA by voices was only observed after voice-face but not voice-name association, ruling out the possibility that FFA activation results from any type of exposure to voices, e.g. an expertise effect [26,27], or any association of a visual stimulus with a voice. As we used only voices of speakers who were unknown to the participants and remained so throughout the experiment, the effect was not driven by contextual knowledge about the speakers [25,38]. Our findings hence provide further indirect corroboration of the functional specialization of the FFA for face processing. They show, however, that input to this region is not exclusively visual, although it is confined to one sensory domain (faces and voices relative to objects). Our data also suggest that FFA functional properties does not merely lie in the processing of physical properties of face stimuli, but are influenced by the processing of faces as multimodal sources, i.e. by early multisensory interactions necessitated by segregation of the sensory organs.

### TVA-FFA Coupling and Speaker Recognition Facilitation

Increase in functional connectivity between voice and face cortical modules was accompanied with facilitation of speaker recognition. Although early TVA-FFA coupling is physiologically plausible and we found no evidence for a neural relay between TVA and FFA after learning, fMRI data alone do not indicate whether voice-face learning truly promotes a direct binding between the two areas, and whether this distributed system constitutes a useful internal sensory representation of the person. In combination with behavioural observations, however, it is possible to distinguish between competing hypotheses. The mere fact that we observe both a strong TVA-FFA coupling and speaker recognition facilitation, when voices have been associated with faces but not with names, rules out the model in which speaker recognition is entirely afforded by a bottom-up process, and TVA-FFA coupling is an irrelevant side effect of speaker recognition (Figure 1A). Physiological aspects of voice processing indicate that speaker identification is, at least in its early stages, a “high uncertainty” task that should not afford immediate person recognition. Speaker identification is not instantaneous, but requires an averaging of the auditory signal over several hundreds of ms during which the uncertainty about speaker identity may translate into neural feed-forward predictive signals. This high degree of uncertainty in voice recognition accordingly produces higher error rates during voice than face recognition [45,46].

The two other proposed models more readily agree with a “high uncertainty” cognitive context. In the first case, voices afford some degree of recognition by bottom-up processing (Figure 1B), which opens access to “contextual” representations of the person possibly distributed across sensory systems; in this scheme, face information is accessed subsequent to a supramodal person recognition stage (a PIN), and all associated information about the speaker should be equally retrieved. We predicted that this configuration would yield speaker recognition facilitation, irrespective of the type of prior associative learning. Participants should equally make use of name and face information, depending on what they previously learned. In the context of ring tone recognition, they should equally retrieve information about brand names and about cell phones. For all types of learning, we did observe a small facilitation effect, amounting to about 5% improvement, in line with previous studies [47]. However, this model does not provide a satisfying framework to the marked gain in speaker recognition following voice-face learning that reached 14% (9% gain relative to voice-name learning). Neither does it fit with the absence of functional coupling between TVA and regions involved in visual word processing (visual word form area, [34]) after voice-name association, and the absence of crossmodal effects after ring tones were paired with visual stimuli. Thus, even if all the associative learning tested here yielded a minor enhancement of sound recognition, this “contextual” facilitation was not underpinned by a strengthening of functional connectivity between cortical regions dedicated to each of the paired stimuli.

Due to physical properties of voice-face pairs, TVA-FFA coupling is very likely to reflect a particular type of multisensory binding, and we therefore proposed a third model in which voices do not directly afford speaker recognition (Figure 1C) but tap into a newly formed, distributed voice-face internal representation making both voice and face information readily available for speaker recognition. The high speaker recognition gain and the changes in functional connectivity, which specifically followed voice-face learning support this model. In the framework of predictive coding, this voice-face representation could enhance speaker recognition by enriching voice templates with distinct visual features, i.e. facial traits that provide a visual sensory account for the voice.

### Stimulus Requirements for Shaping Effective Multimodal Sensory Representations

Models based on predictive coding assume that the brain captures the regularities of the natural world through statistical assessment of the properties of the environment [7,10,48]. Conceptual representations of ecologically valid objects, however, are not reducible to the representation of statistical combination of features [49]. Although all associations in our study offered equivalent statistical connections, voices and faces were distinct because they are by nature not dissociable, i.e. intrinsically connected and providing redundant sensory information. More importantly, vocal amplitude modulations during speech strictly follow mouth, lip, and neck motion. The importance of a common physical source for sounds and visual stimuli in the nature and strength of the coupling elicited by associative learning was addressed by our controls involving ring tones and cell phones. Although these stimuli relate to a single multimodal source, their association is arbitrary. Furthermore, in the videos we used as feedback during learning, biological visual motion did not strictly follow ring tone amplitude modulations. We presented a cell phone held by a hand with one finger reaching for a button to press. Although we thereby ensured the presence of biological motion in both associations, cell phone motion did not follow sound modulations as in speaking faces. The association of ring tones with their visual source did not enhance auditory recognition performance more than ring tone-brand name association, and did not result in specific crossmodal neural effects. We propose that an ecologically valid common source is not sufficient to shape effective multisensory representations, but that sensory redundancy, in particular through the aspect of temporal co-modulation in time, is an important criterion in the formation of an effective multisensory representation. Further studies will have to determine whether the present findings can hold for non-living things. Although this remains speculative, we would predict similar results for non-living multimodal pairs, provided they are sensory redundant (e.g., odors and food, visual and auditory aspects of running water, etc.).

### Person Identity Node and Multisensory Distributed Representations

During learning, both voice-face and voice-name associations were easy but responses were slower for voice-name than voice-face matching. The difference in reaction times (~150 ms) suggests that associating faces with voices involved more direct and automatic mechanisms than associating names with voices, which further supports the distinction between the two models proposed in Figure 1, one (Figure 1C) highlighting a direct binding between voices and faces, and the other (Figure 1B) proposing that other attributes such as names are connected via a person recognition stage, a PIN. Although our findings generally agree with a distributed voice-face sensory representation that augments the amount of sensory information available for speaker recognition, the small recognition facilitation observed after all types of associative learning is compatible with a contribution of a supramodal person identity level, a PIN in the classical sense [12–16], as a means of accessing other contextual information about sounds (Figures 2A and 6). Our results, however, show that access to contextual information through a PIN remains less beneficial to unimodal recognition than direct retrieval of multisensory information.

A detailed analysis of the fMRI data collected during the voice-face and voice-name learning points to a region that met a number of requirements expected for a PIN. The anterior temporal cortex responded more to associating person-related features (voices with either faces or names) than to categorization performed on the same stimulus material. This region also responded to voices in a way that predicted subsequent rapid recognition of an associated visual feature, either a face or a name. Responses to voices in the anterior temporal cortex decreased with reaction time to both face and name visual stimuli. These observations concur to confirm that the anterior temporal pole plays a role during person recognition at a supra-modal level, and agree with our previous observation showing that the left temporal pole responded less in a person with congenital prosopagnosia than in controls both when viewing faces and recognizing familiar speakers [38]. Our results confirm that associating two sensory attributes of the same individual engages this region.

Due to the event related-design employed to monitor learning, we are unable to provide information about functional connectivity of the anterior temporal cortex. Further studies are required to clarify how it interacts with other regions involved in processing person related features. However, activity in the anterior temporal cortex during voice recognition was not enhanced by learning (neither voice-face nor voice-name), nor did we note greater functional connectivity to FFA after learning. One possible reason why learning had no effect on the response in this region could be that it is only engaged during explicit crossmodal matching but not during unimodal recognition. At any rate, the anterior temporal cortex is not essential for the coupling between voice and face areas, but constitutes a more advanced processing stage in person recognition.

### Timing Issues and Potential Generalisation of Current Findings

Several lines of argument indicate that previously formed multisensory representations should not positively influence unimodal perception in all multisensory settings, even when stimuli present sensory redundancy [50]. Redundancy certainly favours multisensory coupling at the neural level, yet perception does not necessarily benefit from this multisensory information. Access to low-level multisensory representations may positively influence unimodal perception when the stimulus presented unimodally engenders perceptual uncertainty that lasts long enough to permit effective multisensory feedback loops to be established. Typically, our finding would not apply to face recognition under optimal visual conditions, although it might help to disambiguate faces, if, for instance, a person is talking via a microphone at a distance. Under normal visual conditions, face recognition is achieved more quickly than voice recognition [45,46]. Accordingly, Joassin et al. [51] report longer reaction times for voice (1,009 ms) than face recognition (853 ms).

The 200-ms difference between face and voice recognition is consistent with the time required to average pitch and spectral envelope information contained in a voice to identify the speaker. This integration duration might set the limit of a time window during which feedback information can be collected and inform perception. Studies on the McGurk effect illustrate that crossmodal effects strongly depend on the uncertainty of the visual signal (i.e. whether the mouth movements are a strong predictor of the corresponding speech, e.g. lip movements producing a “pa” versus the more ambiguous visual “ka” [50]). The authors describe a 200 ms duration temporal integration window for audio-visual McGurk fusion [52] with a stronger fusion effect when the visual stimulus leads by about 70 ms, as it normally happens during audio-visual speech. This physiological time lag offers an appropriate time-window for the generation of predictive signals that influence auditory perception.

A global 200-ms integration window may have a general validity for audio-visual integration (it surely has one for speaker recognition), but the precise time constants of AV integration are expected to be different in the domain of voice/face recognition and in that of speech. In each specific case, it is probably the modality receiving the sparser information (or the signal requiring the longer integration time) that generates the feed-forward signal, and the duration over which feedback information can usefully inform recognition should depend on the duration of the uncertainty. Our findings probably generalize to other multisensory settings provided unimodal information offers enough perceptual uncertainty [48]. We argue that speaker recognition is, by virtue of the long sampling/integration window it requires, accompanied by a high degree of uncertainty and by nature, prone to capitalize on multisensory influence. Further studies are required to address the role played by the duration and amount of uncertainty in controlling multisensory influences during unimodal perception.

### Conclusions

The current experiments point to an important property of perception, which involves the influence of multisensory experience on unimodal auditory perception by engaging visual sensory cortices that are usually co-activated when a natural object is encountered in real life. The key observations are that crossmodal recruitment of visual sensory cortices is behaviourally relevant for unimodal auditory perception, and that multimodal stimulus combinations are neurally represented in the brain as a Gestalt that becomes activated as an ensemble in response to unimodal stimuli. The reactivation and behavioural relevance of multisensory representations is in accord with predictive coding models of brain function in that multimodal Gestalts facilitate the decoding of sparse and in itself insufficient information from a single modality.

## Materials and Methods

### Participants and stimuli.

Twenty-nine right-handed healthy volunteers (11 females) participated in our study after having given their written informed consent along the guidelines of the Ethics committee of the J.W.-Goethe University (Frankfurt/M., Germany). Fourteen of them were included in a group performing a voice-face association and 15 were in a group performing a voice-name association. Behavioural data of one participant performing the voice-name association were lost due to technical failure. All participants additionally underwent a protocol to localize face responsive brain regions.

Each group performed two identical auditory sessions including voice recognition tasks (Parts 1 and 3) carried out before and after a learning protocol (Part 2), which consisted of associating faces or written names with voices (Figure 2). Voices were taken from audio-visual sequences obtained from five speaking actors using a digital video camera (DCR-PC01E, Sony Corporation, Tokyo, Japan; 32-kHz sampling rate, 16-bit resolution). Sequences included semantically neutral, phonologically and syntactically homogeneous sentences (Example: “Die mueden Astronauten verlassen das alte Raumschiff”/“The tired astronauts leave the old space ship”) or sequences of two words (abstract noun and adjective, four syllables in total; Example: “starke Kuerzung”/“strong reduction”). In total, 47 sentences and 111 two-word sequences were recorded from each actor. Only the two-word sequences were used during the experiment while the sentences were presented in the brief auditory familiarization session before the first scan (see below).

The experimental protocol included control conditions for all runs using ring tones of cell phones associated with cell phones or with brand names. Audio-visual sequences of cell phones were recorded with the same material as videos of persons. The videos focused on the hand of an actor holding the cell phone ringing and reaching for a button to press. Please see Supporting Information for examples of the videos (Videos S1 and S2).

All auditory stimuli were post-processed using CoolEdit (Syntrillium Software Corporation, Scottsdale, Arizona, United States) to adjust overall sound pressure.

### Data acquisition.

Functional MRI during voice/ring tone recognition sessions and associative learning was performed on a 1.5-T Siemens Vision scanner (gradient booster, standard head coil), with an echoplanar imaging sequence covering the whole brain (24 slices, 1-mm gap, voxel size 3.44 × 3.44 × 4 mm^3^, Repetition Time = 2.7 s, 145 volumes/participant in the sessions before and after learning, 401 volumes/session/participant in the learning part, and 108 volumes/participant for the face area localizer). Acoustic stimuli were delivered in the MRI scanner with a commercially available high-quality sound system (mr-confon, Magdeburg, Germany, stimuli 80 dB SPL, scanner noise 100 dB, passive attenuation by sound system 40 dB).

### Auditory recognition experiments (Part 1 and Part 3).

Voice and ring tone recognition tasks consisted of 6-min scanning sessions with auditory stimuli presented in a block design. Before the first session, participants were briefly familiarized with the auditory stimuli by passively listening to spoken sentences and ring tones. Participants were not informed of the purpose of the experiment. Conditions were split into four blocks presented in random order within and across conditions. Each block lasted 28 s and contained 16 items (either word sequences or cell phone sounds) of which six were targets to be recognized. Each block was preceded by a fixation cross lasting 9 s. Before each block, participants received the oral instruction to pay attention to voices or ring tones, depending on the condition, and were presented with the target of the ensuing block. As each voice pronounced different words, verbal content could not serve as a cue to identify the targets. Participants were requested to respond to each item with the right hand by pressing one button if it was a target and another button if it was not. Stimuli were presented and responses recorded using Presentation software (http://nbs.neuro-bs.com).

MRI data were analyzed with SPM2 (http://www.fil.ion.ucl.ac.uk/spm). Standard spatial pre-processing (realignment, normalization, and smoothing with a 8-mm Gaussian kernel for group analysis) was performed and statistical parametric maps were generated by modelling the evoked hemodynamic response for the different stimuli as boxcars convolved with a synthetic hemodynamic response function in the context of the general linear model [53]. Population-level inferences concerning BOLD (Blood oxygen level dependant) signal changes between conditions of interest were based on a random effects model that estimated the second level *t* statistics at each voxel for the reference group. Comparisons between groups were performed using a one-way analysis of variance (ANOVA) with correction for non-sphericity. Activity was considered significant at *p* < 0.001 uncorrected if the location was in accordance with prior hypotheses.

Functional connectivity was assessed with PPI analyses using the standard procedure implemented in SPM2 (source code for PPI available at http://www.fil.ion.ucl.ac.uk/spm). We sampled activity in the individual maxima of regions of interest (right precuneus, right parietal and right prefrontal cortices, TVA, and FFA) and probed connectivity resulting from voice-face learning using fixed effects statistics, followed by single participant analyses. To investigate the change in connectivity induced by voice-face association, we contrasted connectivity for voice recognition before and after learning and vice versa.

### Crossmodal learning (Part 2).

Audio-visual associative learning was performed between the two auditory recognition sessions. Prior to scanning the learning part, i.e. immediately after the first auditory session, participants were presented with the correct combination of the voices with the corresponding face/name (ring tones with corresponding cell phone/brand name) twice ([1] voice followed by face/name, [2] voice and face/name simultaneously). This brief familiarization was followed by a short example of the procedure involving each voice and ringtone once (six trials in total). Scanning included two 18-min sessions consisting of trials with voices/ring tones (presented for ~1.3 s), followed by a picture (500 ms) of a face/cell phone (see Figure 2, right column). Participants were requested to indicate via a button press whether the face/cell phone matched the preceding voice/ring tone or not. Visual feedback consisting of a video (~1.3 s) of the speaker speaking or the cell phone ringing followed the picture. In the control group which associated auditory stimuli with written names, the procedure was identical with the exception that the feedback was not a video but the written name that lasted as long as the auditory stimulus (~1.3 s). All participants learned correctly all associations.

We controlled for the sensory characteristics of the stimuli by including a condition in which stimuli were presented in the same order (voice, face\/name, feedback; ring tone, cell phone/name, feedback) during which participants performed a classification task instead of a matching task. They responded to the pictures by indicating whether it was a phone or a face in the sound-video group, or a brand name or a person name in the sound-name group. Stimuli were different from those used in the experimental conditions, i.e., different voices and ring tones.

The learning part of the experiment was analyzed separately as an event-related design. Pre-processing was performed as described above. The same statistical thresholds were applied. We performed two analyses using SPM2. In the first, we analyzed the regions were responses were higher during voice than ring tone recognition in contrast to categorization in both learning groups (Figure 6C). In the second analysis, we identified regions where responses to voices predicted fast and automatic matching of the corresponding picture or name, by probing activation that parametrically followed response speed (Figure 6D). The auditory events followed by a matching picture were modelled together with a regressor corresponding to the response times to the visual stimuli presented after either the voice or the ring tone.

### Functional localizer of the face area.

The visual localizer study involved one 4-min, 30-s run, including two passive viewing conditions: 30-s blocks of faces or objects, alternatively. The stimuli employed were 30 pictures of faces in frontal view and 30 pictures of objects in canonical view. All stimuli were digital 300 × 300 pixels colour pictures. Single stimuli were presented every 600 ms (stimulus on for 450 ms and off for 150 ms). A fixation cross was introduced between the blocks for 16.8 s.

This functional localizer served to establish functional maps of the face area that were used to confirm an overlap between the response to faces and the crossmodal effect in response to voices (Figure 3). Data for the face area localizer were pre-processed and analyzed as the auditory recognition experiments described above. The analysis was based on a random effects model and the ROI was defined using SPM2 and MarsBaR ROI toolbox (http://marsbar.sourceforge.net). For the sound-video group the FFA was located at 45, −45, −24 and contained 19 voxels at *p* < 0.05 uncorrected. For both groups together it was located at 45, −45, −27 and contained 34 voxels at *p* < 0.001.

## Supporting Information

Protocol S1 Responses to the Questionnaire (26 KB DOC)

Video S1 Example of the Videos Used for Voice-Face Learning (902 KB MPG)

Video S2 Example of the Videos Used for Ring Tone-Cell Phone Learning (1.1 MB AVI)

## Abbreviations

ANOVA: analysis of variance
BA: Brodmann area
BOLD: blood oxygen level dependent
FFA: fusiform face area
fMRI: functional magnetic resonance imaging
PIN: person identity node
PPI: psychophysiological interaction
ROI: region of interest
TVA: temporal voice area

## References

[pbio-0040326-b001] Kamachi M, Hill H, Lander K, Vatikiotis-Bateson E (2003). “Putting the face to the voice”: Matching identity across modality. Curr Biol.

[pbio-0040326-b002] Smith DRR, Patterson RD, Turner R, Kawahara H, Irino T (2005). The processing and perception of size information in speech sounds. J Acoust Soc Am.

[pbio-0040326-b003] Yehia H, Rubin P, Vatikiotis-Bateson E (1998). Quantitative association of vocal-tract and facial behavior. Speech Communication.

[pbio-0040326-b004] Lachs L, Pisoni DB (2004). Specification of crossmodal source information in isolated kinematic displays of speech. J Acoust Soc Am.

[pbio-0040326-b005] Friston K (2005). A theory of cortical responses. Philos Trans R Soc Lond B Biol Sci.

[pbio-0040326-b006] Murray SO, Schrater P, Kersten D (2004). Perceptual grouping and the interactions between visual cortical areas. Neural Netw.

[pbio-0040326-b007] Rao RP, Ballard DH (1999). Predictive coding in the visual cortex: A functional interpretation of some extra-classical receptive-field effects. Nat Neurosci.

[pbio-0040326-b008] Wolpert DM, Ghahramani Z, Jordan MI (1995). An internal model for sensorimotor integration. Science.

[pbio-0040326-b009] Yuille A, Kersten D (2006). Vision as Bayesian inference: Analysis by synthesis?. Trends Cogn Sci.

[pbio-0040326-b010] Knill D, Kersten D, Yuille A, Knill D, Richards W (1998). Introduction: A Bayesian formulation of visual perception. Perception as Bayesian Inference.

[pbio-0040326-b011] Mesulam MM (1998). From sensation to cognition. Brain.

[pbio-0040326-b012] Ellis HD, Jones DM, Mosdell N (1997). Intra- and inter-modal repetition priming of familiar faces and voices. Br J Psychol.

[pbio-0040326-b013] Neuner F, Schweinberger SR (2000). Neuropsychological impairments in the recognition of faces, voices, and personal names. Brain Cogn.

[pbio-0040326-b014] Young AW, Burton AM (1999). Simulating face recognition: Implications for modelling cognition. Cogn Neuropsych.

[pbio-0040326-b015] Bruce V, Young A (1986). Understanding face recognition. Br J Psychol.

[pbio-0040326-b016] Burton AM, Bruce V, Johnston RA (1990). Understanding face recognition with an interactive activation model. Br J Psychol.

[pbio-0040326-b017] Snowden JS, Thompson JC, Neary D (2004). Knowledge of famous faces and names in semantic dementia. Brain.

[pbio-0040326-b018] Gainotti G, Barbier A, Marra C (2003). Slowly progressive defect in recognition of familiar people in a patient with right anterior temporal atrophy. Brain.

[pbio-0040326-b019] McIntosh AR, Cabeza RE, Lobaugh NJ (1998). Analysis of neural interactions explains the activation of occipital cortex by an auditory stimulus. J Neurophysiol.

[pbio-0040326-b020] Calvert GA, Bullmore ET, Brammer MJ, Campbell R, Williams SC (1997). Activation of auditory cortex during silent lipreading. Science.

[pbio-0040326-b021] Calvert GA, Campbell R (2003). Reading speech from still and moving faces: The neural substrates of visible speech. J Cogn Neurosci.

[pbio-0040326-b022] Ghazanfar AA, Maier JX, Hoffman KL, Logothetis NK (2005). Multisensory integration of dynamic faces and voices in rhesus monkey auditory cortex. J Neurosci.

[pbio-0040326-b023] Kanwisher N, McDermott J, Chun MM (1997). The fusiform face area: A module in human extrastriate cortex specialized for face perception. J Neurosci.

[pbio-0040326-b024] Belin P, Zatorre RJ, Lafaille P, Ahad P, Pike B (2000). Voice-selective areas in human auditory cortex. Nature.

[pbio-0040326-b025] von Kriegstein K, Kleinschmidt A, Sterzer P, Giraud AL (2005). Interaction of face and voice areas during speaker recognition. J Cogn Neurosci.

[pbio-0040326-b026] Gauthier I, Skudlarski P, Gore JC, Anderson AW (2000). Expertise for cars and birds recruits brain areas involved in face recognition. Nat Neurosci.

[pbio-0040326-b027] Bukach CM, Gauthier I, Tarr MJ (2006). Beyond faces and modularity: The power of an expertise framework. Trends Cogn Sci.

[pbio-0040326-b028] Murray MM, Foxe JJ, Wylie GR (2005). The brain uses single-trial multisensory memories to discriminate without awareness. Neuroimage.

[pbio-0040326-b029] Gorno-Tempini ML, Price CJ, Josephs O, Vandenberghe R, Cappa SF (1998). The neural systems sustaining face and proper-name processing. Brain.

[pbio-0040326-b030] Leveroni CL, Seidenberg M, Mayer AR, Mead LA, Binder JR (2000). Neural systems underlying the recognition of familiar and newly learned faces. J Neurosci.

[pbio-0040326-b031] Nakamura K, Kawashima R, Sugiura M, Kato T, Nakamura A (2001). Neural substrates for recognition of familiar voices: A PET study. Neuropsychol.

[pbio-0040326-b032] Ishai A, Haxby JV, Ungerleider LG (2002). Visual imagery of famous faces: Effects of memory and attention revealed by fMRI. Neuroimage.

[pbio-0040326-b033] Mechelli A, Price CJ, Friston KJ, Ishai A (2004). Where bottom-up meets top-down: Neuronal interactions during perception and imagery. Cereb Cortex.

[pbio-0040326-b034] Cohen L, Dehaene S, Naccache L, Lehericy S, Dehaene-Lambertz G (2000). The visual word form area: Spatial and temporal characterization of an initial stage of reading in normal subjects and posterior split-brain patients. Brain.

[pbio-0040326-b035] Friston KJ, Buechel C, Fink GR, Morris J, Rolls E (1997). Psychophysiological and modulatory interactions in neuroimaging. Neuroimage.

[pbio-0040326-b036] Sheffert SM, Olson E (2004). Audiovisual speech facilitates voice learning. Percept Psychophys.

[pbio-0040326-b037] Tsukiura T, Mochizuki-Kawai H, Fujii T (2006). Dissociable roles of the bilateral anterior temporal lobe in face-name associations: An event-related fMRI study. Neuroimage.

[pbio-0040326-b038] von Kriegstein K, Kleinschmidt A, Giraud AL (2006). Voice recognition and crossmodal responses to familiar speakers' voices in prosopagnosia. Cereb Cortex.

[pbio-0040326-b039] Ghazanfar AA, Schroeder CE (2006). Is neocortex essentially multisensory?. Trends Cogn Sci.

[pbio-0040326-b040] Giard MH, Peronnet F (1999). Auditory-visual integration during multimodal object recognition in humans: A behavioral and electrophysiological study. J Cogn Neurosci.

[pbio-0040326-b041] Cappe C, Barone P (2005). Heteromodal connections supporting multisensory integration at low levels of cortical processing in the monkey. Eur J Neurosci.

[pbio-0040326-b042] Falchier A, Clavagnier S, Barone P, Kennedy H (2002). Anatomical evidence of multimodal integration in primate striate cortex. J Neurosci.

[pbio-0040326-b043] Fu KM, Johnston TA, Shah AS, Arnold L, Smiley J (2003). Auditory cortical neurons respond to somatosensory stimulation. J Neurosci.

[pbio-0040326-b044] Schroeder CE, Lindsley RW, Specht C, Marcovici A, Smiley JF (2001). Somatosensory input to auditory association cortex in the macaque monkey. J Neurophysiol.

[pbio-0040326-b045] Hanley JR, Smith ST, Hadfield J (1998). I recognise you but I can't place you. An investigation of familiar-only experiences during tests of voice and face recognition. Quarterly J Exp Psychol.

[pbio-0040326-b046] Hanley JR, Turner JM (2000). Why are familiar-only experiences more frequent for voices than for faces?. Q J Exp Psychol A.

[pbio-0040326-b047] Lehmann S, Murray MM (2005). The role of multisensory memories in unisensory object discrimination. Brain Res Cogn Brain Res.

[pbio-0040326-b048] Ernst MO, Banks MS (2002). Humans integrate visual and haptic information in a statistically optimal fashion. Nature.

[pbio-0040326-b049] Prasada S, Dillingham EM (2006). Principled and statistical connections in common sense conception. Cognition.

[pbio-0040326-b050] van Wassenhove V, Grant KW, Poeppel D (2005). Visual speech speeds up the neural processing of auditory speech. Proc Natl Acad Sci U S A.

[pbio-0040326-b051] Joassin F, Maurage P, Bruyer R, Crommelinck M, Campanella S (2004). When audition alters vision: An event-related potential study of the cross-modal interactions between faces and voices. Neurosci Lett.

[pbio-0040326-b052] van Wassenhove V, Grant KW, Poeppel D (2006). Temporal window of integration in auditory-visual speech perception. Neuropsychologia.

[pbio-0040326-b053] Friston KJ, Holmes AP, Worsley KJ, Poline JP, Frith CD (1995). Statistical parametric maps in functional imaging: A general linear approach. Human Brain Mapping.

