# Supplementary material for: Implicit Multisensory Associations Influence Voice Recognition
Source: PLoS Biol. 2006 Sep 26;4(10):e326. doi: 10.1371/journal.pbio.0040326 (PMC1570760; doi:10.1371/journal.pbio.0040326)
Supplement: Supplementary file 1 [file pbio.0040326.sd001.doc]

**Implicit Multisensory Associations Influence Unimodal Voice Recognition**

Running head: Multisensory Influences on Unimodal Recognition

Katharina von Kriegstein^1,3^* and Anne-Lise Giraud^1,2^

**Supporting Information**

**Responses to the questionnaire**

After scanning we asked the subjects to answer the following questions:

1- Did you imagine faces or objects to the voices and sounds in the sessions 1 and/or 4 (before/after learning)?

2- Did you name voices or sounds in the sessions 1 and/or 4 (before/after learning)?

8 of the fourteen subjects in the face group reported that they imagined fantasy faces even before learning and 12 subject said that they had the face corresponding to the voice in mind after learning. In the voice-name group only 4 of the fifteen subjects said that they had imagined people before and after learning of the names.
